# Pseudo-magnetic field-induced slow carrier dynamics in periodically strained graphene

**DOI:** 10.1038/s41467-021-25304-0

**Published:** 2021-08-24

**Authors:** Dong-Ho Kang, Hao Sun, Manlin Luo, Kunze Lu, Melvina Chen, Youngmin Kim, Yongduck Jung, Xuejiao Gao, Samuel Jior Parluhutan, Junyu Ge, See Wee Koh, David Giovanni, Tze Chien Sum, Qi Jie Wang, Hong Li, Donguk Nam

**Affiliations:** 1 School of Electrical and Electronic Engineering, Nanyang Technological University, Singapore, Singapore; 2 School of Mechanical and Aerospace Engineering, Nanyang Technological University, Singapore, Singapore; 3 Division of Physics and Applied Physics, School of Physical and Mathematical Sciences, Nanyang Technological University, Singapore, Singapore

**Keywords:** Optical properties and devices, Optical properties and devices

## Abstract

The creation of pseudo-magnetic fields in strained graphene has emerged as a promising route to investigate intriguing physical phenomena that would be unattainable with laboratory superconducting magnets. The giant pseudo-magnetic fields observed in highly deformed graphene can substantially alter the optical properties of graphene beyond a level that can be feasible with an external magnetic field, but the experimental signatures of the influence of such pseudo-magnetic fields have yet to be unveiled. Here, using time-resolved infrared pump-probe spectroscopy, we provide unambiguous evidence for slow carrier dynamics enabled by the pseudo-magnetic fields in periodically strained graphene. Strong pseudo-magnetic fields of ~100 T created by non-uniform strain in  graphene on nanopillars are found to significantly decelerate the relaxation processes of hot carriers by more than an order of magnitude. Our findings offer alternative opportunities to harness the properties of graphene enabled by pseudo-magnetic fields for optoelectronics and condensed matter physics.

## Abstract (web summary)

The effect of strain-induced pseudo-magnetic fields on the optical properties of graphene has not been experimentally explored yet. Here, pseudo-magnetic fields reaching values of 100 T are shown to increase by more than an order of magnitude the relaxation lifetime of hot carriers in periodically strained graphene.

## Introduction

Since its discovery in 2004^1,2^, graphene has continued to revolutionize a wide variety of research fields including physics, electronics, and photonics, to name a few^3^. Among the surprising properties is its exceptional mechanical strength^4^, which has spurred intense research activity on strain engineering of graphene^5^. Approximately a decade ago, the creation of gauge fields in graphene by harnessing non-uniform strain began attracting considerable attention as a potential route towards realizing previously unattainable physical properties in graphene^6^. Particularly, a theoretical study predicted that a uniquely designed strain in graphene could induce pseudo-magnetic fields that would allow electrons to behave as if they were subjected to a strong real magnetic field^7^. The strength of pseudo-magnetic fields in strained graphene can be orders of magnitude higher than the strength of external magnetic fields generated by superconducting magnets^8–10^, thus prompting researchers to experimentally verify the existence of such pseudo-magnetic fields. Since then, scanning tunneling spectroscopy (STS) measurements have repeatedly confirmed that pseudo-magnetic fields in mechanically deformed graphene can reach up to a few hundred T^11–16^. These giant pseudo-magnetic fields can form significant energy gaps^7^ and substantially modify optical transitions in the same manner as an external magnetic field^17–20^, but to a previously unattainable extent due to the high strength of pseudo-magnetic fields. However, the influence of pseudo-magnetic fields on the optical properties of graphene has not yet been experimentally verified.

In this article, we present an experimental observation of the effect of pseudo-magnetic fields on hot carrier relaxation processes. The strain engineering platform employed in this study allows creating a non-uniform strain distribution with a maximum strain field of ~3.5% that is higher than other reported values for sustainable strain in graphene. The magnitude of the induced pseudo-magnetic fields is confirmed to be ~100 T via rigorous tight-binding simulations combined with the elasticity theory. Using time-resolved infrared pump–probe spectroscopy combined with theoretical modeling based on many-body interactions, we experimentally confirm that the induced pseudo-magnetic fields in our strained graphene system can decelerate the hot carrier relaxation processes by more than an order of magnitude.

## Results

### Design of strain-engineered graphene nanostructure array

**Fig. 1:**
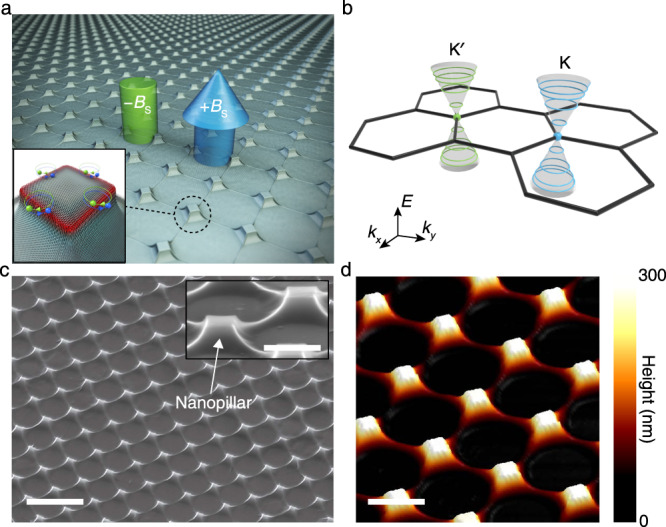
Design of a strain-engineered graphene nanostructure array. **a** Schematic illustration of a strained graphene nanopillar array possessing pseudo-magnetic fields of positive (+*B*_S_, blue arrow) and negative (−*B*_S_, green arrow) signs. Inset: magnified view of a single strained graphene nanopillar structure. Non-uniform tensile strain at the edges of the nanopillar induces pseudo-magnetic fields of opposite signs (−*B*_S_ and +*B*_S_) in the two valleys of the graphene band structure, thus forcing electrons in the K and K′ valleys to circulate in the opposite directions. **b** Schematic illustration showing Landau quantization at the K and K′ points in the first Brillouin zone of graphene in the presence of pseudo-magnetic fields. **c** Tilted-view SEM image of a strained graphene nanopillar array. Scale bar, 2 μm. Inset: magnified SEM image. Scale bar, 500 nm. **d** AFM topography of the strained graphene nanopillar array. Scale bar, 1 μm.

A typical strain-engineered graphene nanostructure array and the key concept for the generation of pseudo-magnetic fields are illustrated in Fig. 1a, b. A monolayer graphene sheet was forced to conform to the topography of the nanopillar array using capillary force (see Methods and Supplementary Note 1 for the detailed fabrication procedure)^21–25^. Graphene’s excellent mechanical property^4^ allows the accumulation of a large tensile strain at the edges of nanopillars^10^. The resultant non-uniform strain distribution near the edges of nanopillars can generate strong pseudo-magnetic fields, which force electrons to move in a circular motion as if they are under a strong external magnetic field^7,8^. Due to the preserved global time-reversal symmetry under the influence of strain-induced pseudo-magnetic fields, electrons in the K and K′ valleys experience pseudo-magnetic fields of opposite signs, thus circulating in opposite directions^12^. This cyclotron motion of the charge carriers corresponds to the creation of pseudo-Landau levels (Fig. 1b), which enables us to observe substantially modified optical transitions in a strained graphene device. Figure 1c, d shows scanning electron microscopy (SEM) and atomic force microscopy (AFM) images of the strained graphene device used in this study. The structural analyses reveal that graphene is strongly deformed at the sharp corners and edges without showing any signature of fracture across the entire array.

### Formation of pseudo-magnetic fields in strained graphene

**Fig. 2:**
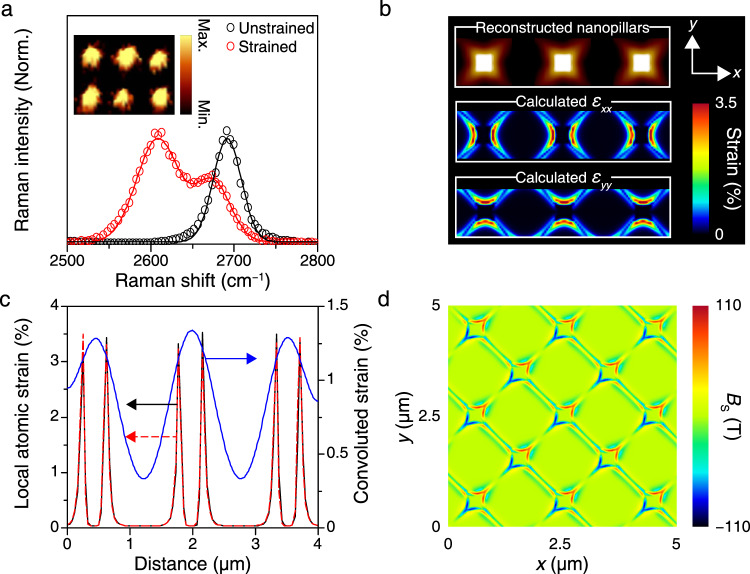
Formation of strong pseudo-magnetic fields in highly strained graphene. **a** Raman spectra of unstrained (black) and strained (red) graphene. Symbols are measurement data; curves are fitting data. Inset: Two-dimensional Raman mapping data plotting the 2D peak frequency of a 3 × 2 strained graphene nanopillar array. **b** The topographic image of the reconstructed a 3 × 1 strained graphene nanopillar array (top panel) and corresponding local strain distributions along the *x* (${{\epsilon }}_{xx}$, middle panel) and *y* (${{\epsilon }}_{yy}$, bottom panel) directions. The brightest and darkest regions in the topographic image indicate 300-nm and 0-nm heights of structure, respectively. **c** Cross-section of the strain profile showing local atomic strain for ${{\epsilon }}_{xx}$ (black solid line) and ${{\epsilon }}_{yy}$ (red dashed line), and convoluted strain distribution (blue solid line). **d** Pseudo-magnetic fields distribution in strained graphene nanopillar array.

Figure 2a presents the Raman spectrum measured for strained graphene on the nanopillar (red) (see Methods for more details on Raman spectroscopy); for comparison, the Raman spectrum for the unstrained control device (black) is also presented. Two-dimensional Raman mapping was performed to confirm the highly periodic nature of our strained nanopillar array (Fig. 2a, inset). The measured 2D Raman peak shift of ~85.2 cm^−1^ is larger than other reported values for deformed graphene on nanostructured substrates^22–25^, demonstrating the excellence of our strain engineering platform (see Supplementary Note 2 for more details on G and 2D peak shift analyses). Figure 2b shows the local strain distribution based on the experimentally obtained topographic information (see Supplementary Note 3 for the strain calculation). To calculate the local strain distribution, we employed a reconstructed 3 × 1 strained graphene nanopillar array (top panel), in which the brightest and the darkest regions correspond to the 300-nm and 0-nm heights of the structure, respectively. The lateral size of nanopillars is approximately 300 nm (Supplementary Fig. 5). Strong structural deformation near the sharp corners and edges of the nanopillars generated a substantial tensile strain of up to ~3.5% at the atomic scale. By decomposing the strain distribution along the *x* and *y* directions as shown in the middle (${{\epsilon }}_{xx}$) and bottom (${{\epsilon }}_{yy}$) panels, respectively, we determined that ${{\epsilon }}_{yy}$ is nearly absent where ${{\epsilon }}_{xx}$ is maximum, indicating that our strained graphene nanopillar is largely under uniaxial strain. A maximum experimental strain value of 1.3% was derived from the measured Raman shift (Fig. 2a) by using a strain-shift coefficient^26^ (see Methods). This discrepancy between the simulated and measured maximum strain values is attributable to the inherent resolution limit of the Raman measurement with the diffraction-limited laser spot size^21^. By performing convolution on the simulated atomic-scale strain distribution by using a two-dimensional Gaussian corresponding to the spot size of the laser beam^21^, a realistic strain distribution that could be optically measured on the nanopillar was deduced, yielding a maximum strain value of 1.32% (Fig. 2c). A comparison between the measured strain from the Raman shift and the convoluted strain obtained from atomic-scale simulations revealed excellent quantitative agreement. It should be noted that a clear strain variation between the edges and the central part of a single nanopillar can also be observed (Supplementary Fig. 6) provided that the size of a nanopillar is larger than the theoretical spatial resolution limit (~361 nm) of our Raman system (see Methods for the calculation of theoretical spatial resolution limit). The spatial distribution of the pseudo-magnetic fields (*B*_S_) at the atomic scale (Fig. 2d) was obtained using a well-developed method based on the tight-binding simulation^6–9,11,12,27^, which couples the Dirac equation to the deformed graphene surface to obtain the following relationship between strain fields and gauge fields:

1 $${A}_{x}=\frac{\beta }{2{a}_{0}}({\epsilon }_{xx}-{\epsilon }_{yy}),\,{A}_{y}=\frac{\beta }{2{a}_{0}}(-2{{\epsilon }}_{xy}),$$

where *β* is a constant (~3), *a*_0_ is the nearest carbon–carbon bond length (~0.14 nm), and $\epsilon$ is a 2 × 2 strain tensor. This simulation method has successfully replicated the atomic-scale experimental pseudo-magnetic field distribution measured by STS^11,12,16^, thus ensuring the reliability of our simulation (see Supplementary Note 4 for more details on the pseudo-magnetic field simulation). As shown in Fig. 2d, the pseudo-magnetic fields reach up to approximately 100 T near the sharp edges and corners that host the largest deformation and the steepest strain gradient (Fig. 2b), which is largely consistent with ref. ^10^.

### Pseudo-magnetic field-induced slow carrier dynamics

**Fig. 3:**
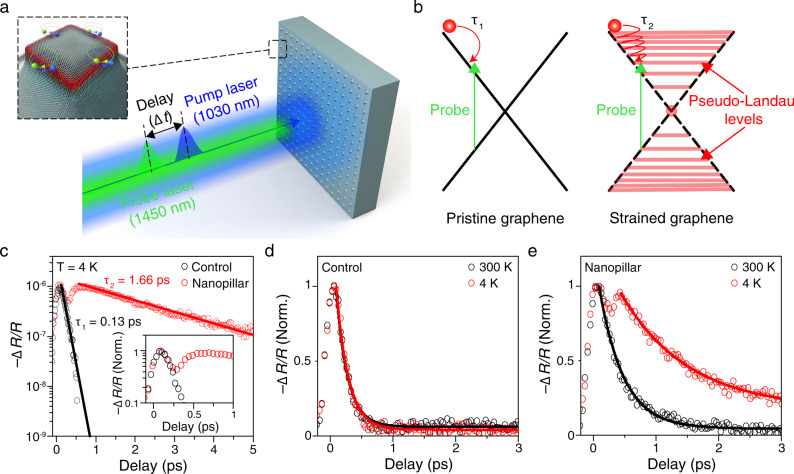
Pseudo-magnetic field-induced ultra-slow carrier dynamics. **a** Schematic illustration of our femtosecond pump–probe measurement setup. Pump pulses (blue) excite charge carriers and probe pulses (green) are used to monitor the sample responses at different delay times (Δ*t*) after the pump pulses arrive at the sample. Left inset: magnified image of a single nanopillar structure. **b** Schematic illustration of relaxation process of photoexcited carriers in pristine graphene with massless Dirac cones (left) and strained graphene attaining pseudo-Landau levels (right). The formation of pseudo-Landau levels can significantly decelerate the relaxation process, resulting in a longer decay time in strained graphene (${\tau }_{1}$ « ${\tau }_{2}$). **c** Measured reflection change as a function of the delay time on control (black) and nanopillar (red) samples. Symbols are measurement data; lines are fitting data for the decay region. The decay times of both samples are extracted from fitting data; ${\tau }_{1}$ = 0.13 ps (Control) and ${\tau }_{2}$ = 1.66 ps (Nanopillar). Inset: Normalized reflection change of both samples from −0.15 to 1 ps. **d**, **e** Normalized reflection change of control (**d**) and nanopillar (**e**) samples measured at 300 K (black) and 4 K (red). Symbols are measurement data; lines are fitting data for the decay region.

To study the influence of pseudo-magnetic fields on the optical properties of graphene, we performed ultrafast pump–probe spectroscopy (see Methods for measurement details). Figure 3a presents a conceptual illustration of our femtosecond pump–probe measurement setup. The devices were pumped by femtosecond laser pulses while time-delayed probe pulses were used to monitor the photo-induced change in the reflection spectrum (Δ*R*/*R*), which is directly proportional to the absorption in graphene for monolayer devices^28–30^. The measurement was performed at 4 K, unless stated otherwise, and low pump fluence (<1 mJ cm^−2^) was used to avoid any heating effect (see Methods for details). The probe pulse spot size was approximately 10 × 10 μm^2^, thus allowing us to probe a large number (>16) of highly identical nanopillars simultaneously for a large effective area possessing sizable pseudo-magnetic fields. The strength of pseudo-magnetic fields is negligible in graphene placed on flat surfaces (Fig. 2d) where there is little out-of-plane lattice distortion (Fig. 2b), which is predominantly responsible for building up pseudo-magnetic fields^12^. Within the area of investigation governed by the probe beam size, therefore, pristine graphene with massless Dirac cones (Fig. 3b, left) existed simultaneously with strained graphene in which pseudo-Landau levels were attained (Fig. 3b, right), and both contributed to the measured data presented in Fig. 3c, e.

To better understand the carrier dynamics of the device on nanopillars possessing pseudo-magnetic fields, we first performed control experiments involving the same monolayer graphene sheet on an entirely flat surface without nanopillars. In pristine graphene with massless Dirac cones (Fig. 3b, left), the optically excited carriers by pump pulses (*λ*_pump_ ~1030 nm) relax to the lower energy states rapidly within a few tens of femtoseconds through electron–electron scattering, thus inhibiting the absorption of probe pulses at the corresponding energy (*λ*_probe_ ~1450 nm) due to Pauli blocking^30–43^. This decreased absorption in the control device was reflected in the rapid rise of the negative reflection change (−Δ*R*/*R*) (Fig. 3c, black empty circles). Subsequently, the number of charge carriers in the particular energy state rapidly reduces within 100–200 fs through electron–electron and electron–optical phonon scattering processes^30,31,36–39^, and this was revealed by the rapid decay of −Δ*R*/*R*. The evolution of these fast relaxation processes occurred within the temporal resolution of our probe pulse (~230 fs). A subsequent slow relaxation process with a very weak intensity via the emission of acoustic phonons^40^ can also be observed in our sample, but only at higher pump fluences. In this study, we focused on the initial rapid carrier relaxation processes because of their importance in various crucial device applications^19,35,44–49^.

The response of the nanopillar device is also presented in the same figure (Fig. 3c, red empty circles). Both control and nanopillar samples were fabricated on the same substrate (i.e., Al_2_O_3_) to avoid any unexpected effects from substrate phonons^50,51^, which may influence the carrier dynamics. Immediately after pump excitation, −Δ*R*/*R* of the nanopillar device quickly rose and decayed on the same time scale as that of the control device. Surprisingly, −Δ*R*/*R* rose again very slowly until ~700 fs, followed by a slow decay with a time constant of ~1.66 ps. To illustrate the two distinct regimes, a detailed view over a shorter time range is shown in the inset of Fig. 3c. To understand the origins of these regimes, we turn to Fig. 3b. As mentioned earlier, both pristine graphene and strained graphene with pseudo-magnetic fields contributed to the signal from the nanopillar device. The rapid change in −Δ*R*/*R* in the rapid regime can be clearly attributed to pristine graphene with fast carrier relaxation processes (Fig. 3b, left). Unlike pristine graphene, the relaxation process in strained graphene can be markedly influenced by pseudo-Landau levels (Fig. 3b, right). First, the initial electron–electron scattering of the optically excited carriers in higher energy states corresponding to the pump energy can be suppressed in the presence of pseudo-Landau levels, which substantially slow down the filling process in the lower energy states corresponding to the probe energy. The pseudo-Landau levels also impede subsequent electron–electron and electron–optical phonon scattering processes, resulting in a slow depletion of hot carriers from the probe energy states. The hypothesized carrier dynamics of the strained graphene with pseudo-Landau levels is unambiguously captured in the temporal evolution of −Δ*R*/*R* in the slow regime. This slow carrier dynamics in the presence of pseudo-Landau levels can be explained by the significant reduction of phase space for scattering processes, which has also been experimentally observed in pristine graphene^52^ and other materials^18,53^ but only in the presence of an external magnetic field.

To further support the role of pseudo-Landau levels in producing these effects, we investigated the temperature-dependent carrier dynamics of both the control and the nanopillar devices. Although there was no appreciable change in the carrier dynamics at different lattice temperatures in the control device (Fig. 3d), the nanopillar device exhibited substantially slower carrier dynamics at 4 K with respect to that at 300 K (Fig. 3e). Previous studies have confirmed that the initial fast relaxation dynamics of pristine graphene is insensitive to the lattice temperature due to the efficient electron–electron and electron–optical phonon processes^30,36,52,54^. In contrast, strong temperature dependence of the carrier dynamics was observed in Landau-quantized graphene with an external magnetic field, and this was attributed to the significantly reduced electron–electron scattering at low temperatures in the presence of Landau quantization^52^. The contrasting temperature dependence of the carrier dynamics, as shown in Fig. 3d, e, is highly consistent with the previously observed contrasting phenomena in pristine graphene and Landau-quantized graphene, thus providing another strong evidence that points to the formation of pseudo-Landau levels in the nanopillar device. It should be noted that the carrier relaxation process is influenced by the extent of broadening of pseudo-Landau levels, which is dependent on lattice temperature rather than electronic temperature^38,55^. While the electronic temperature may rise significantly during the pump–probe experiments, the lattice temperature remains largely unchanged^38^, thereby allowing the observation of a significant slow-down of the carrier relaxation process in the nanopillar devices. We also observed that the decay becomes slower at lower pump fluences due to the reduced electron–electron scattering (Supplementary Fig. 7).

### Theoretical modeling of slow carrier dynamics

Optical Bloch equations based on many-body interactions were used to quantitatively understand the relaxation dynamics of charge carriers under the influence of pseudo-magnetic fields. The main many-body interaction process considered in the modeling was the electron–electron scattering that is the dominant relaxation mechanism in Landau-quantized graphene^52^. This electron–electron scattering can be very efficient at the three energetically lowest Landau levels because the energy levels are equidistant, thus fulfilling energy conservation^33^. Since our study focuses on the carrier dynamics in the higher Landau levels with non-equidistant energy spacing, it is difficult to fulfill the energy conservation, thus resulting in significant suppression of carrier relaxation^53^. We model the dynamics of the charge carriers for the initial excitation to the pump energy states and the filling-in to the probe energy states as illustrated in Fig. 3b. Here, according to the electrical characteristic of our monolayer graphene sheet shown in Supplementary Fig. 8, we assumed that the doping level of graphene is close to undoped^56^. The subsequent depletion of the charge carriers out of the probe energy states was also considered (see Supplementary Note 6 for the details on theoretical modeling). The carrier dynamics can be well captured by the Boltzmann-like scattering equation^57^, which is the effective theoretical tool to study the thermalization of hot carriers after pump excitations (see Supplementary Note 7 for more details).

**Fig. 4:**
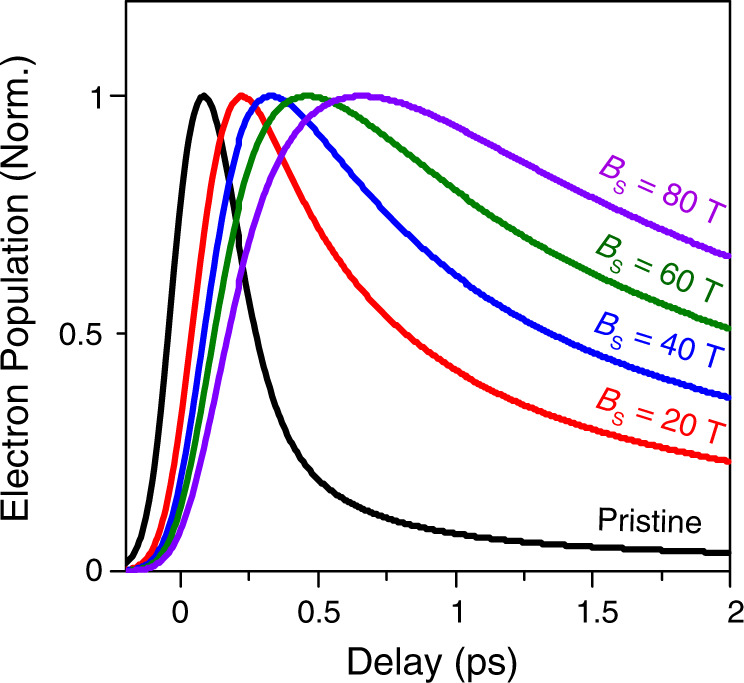
Theoretical modeling of carrier dynamics under the influence of pseudo-magnetic fields. Calculated electron population in the probe energy states as a function of the delay time for strained graphene with different pseudo-magnetic fields (*B*_S_) of 0 T (black), 20 T (red), 40 T (blue), 60 T (green), and 80 T (purple). All graphs are normalized. With the increased pseudo-magnetic field intensity, the rise time increases significantly up to 800 fs for *B*_S_ = 80 T.

Figure 4 presents the calculated electron population in the probe energy states as a function of the delay time. The electron population is directly proportional to the decreased absorption and the negative reflection change (−Δ*R*/*R*) (Fig. 3c–e) because the occupied probe energy states suppress the absorption of probe pulses due to Pauli blocking as explained earlier. This result reveals a clear slow-down of the carrier relaxation processes under the influence of strong pseudo-magnetic fields, which is in reasonable agreement with our experimental results as shown in Fig. 3c, e. Supplementary Fig. 9 shows that the first and second peaks in Fig. 3c are well matched to the calculated carrier dynamics for pseudo-magnetic field intensities of 0 T and ~25 T, respectively. The calculated average pseudo-magnetic field intensity for the strained part is ~25 T (Supplementary Fig. 9a), thus providing an excellent quantitative agreement between experiments and simulations. The slight discrepancy between the theoretical and experimental results may be attributed to the non-uniform pseudo-magnetic field distribution.

## Discussion

In summary, we have presented an experimental observation of the effect of pseudo-magnetic fields on the carrier relaxation processes in highly strained graphene. Our periodic nanopillar structure array achieves strong pseudo-magnetic fields of up to 100 T, which play a major role in enabling the observation of an extended hot carrier relaxation lifetime by more than an order of magnitude. The pseudo-magnetic field-induced slow carrier dynamics in highly strained graphene poses crucial implications towards the realization of a new class of graphene-based optoelectronic devices, including pseudo-Landau level lasers^44^ and highly efficient hot electron harvesting devices^35^. The light emission^45,58^ and population inversion properties^46^ in graphene can be improved drastically by slowing down the hot carrier relaxation processes through pseudo-magnetic fields.

One constraint of our nanopillar structure is that the obtained pseudo-magnetic field is spatially varying, thus inhibiting us to study the isolated effect of specific pseudo-magnetic field strength on the carrier dynamics. In Supplementary Fig. 10, we propose a practical graphene nanowire design that can achieve uniform pseudo-magnetic fields over a large area^8^. This design with uniform pseudo-magnetic fields may allow addressing individual Landau level transitions under a specific pseudo-magnetic field strength. Another disadvantage of graphene under pseudo-magnetic fields is that energetically degenerate transitions cannot be optically selected via circularly polarized radiation, while, for pristine graphene under real magnetic fields, circularly polarized radiation can selectively excite only one of two degenerate transitions^33^. Instead, we propose a possibility to harness a valley degree of freedom in strained graphene. As shown in Supplementary Fig. 12, circularly polarized radiation may allow distinguishing valley-specific electrons in strained graphene under uniform pseudo-magnetic fields, thus implying a potential for graphene valleytronics^48,49^.

It is also worth noting that for the cooling process at a longer timescale, optical phonons hardly fulfill the energy conservation and the momentum conservation in Landau-quantized graphene systems in external magnetic fields^59^. In contrast, our nanopillar devices with highly strained graphene under pseudo-magnetic fields may possess disorder-assisted relaxation channels (i.e., supercollisions), which may dominate the carrier cooling process at a longer time scale^59^. A further study on the effect of symmetry-breaking supercollisions on the carrier dynamics at a longer timescale may provide further insights towards building pseudo-Landau-quantized graphene devices. By presenting experimental evidence of the effect of pseudo-magnetic fields, our finding offers a new landscape of opportunities towards creating pseudo-magnetic field-based graphene optoelectronic devices.

## Methods

### Device fabrication

A nanostructured substrate was fabricated by buffered oxide etch (BOE)-based wet-etching process. For an etching mask (an array with 1-μm diameter holes formed at 1.6-μm intervals), we patterned polymethyl methacrylate resist (950 PMMA A6, MICROCHEM) on SiO_2_/Si substrate using Raith e-line e-beam lithography system built in field-emission SEM (JEOL JSM-7600F). The substrate was then dipped in BOE (12.5% HF, 87.5% NH_4_F) for 3 min 30 s at room temperature, followed by atomic layer deposition (ALD) for depositing a 20-nm Al_2_O_3_ layer on the entire substrate. The fabricated nanopillars have the lateral size of 300 nm (see Supplementary Fig. 5). Graphene monolayer was transferred onto the nanostructured substrate via wet-transfer technique. To make a tight adhesion between graphene and nanostructures, capillary force-induced drying technique was employed^21^. Further details on the fabrication process are provided in Supplementary Note 1.

### Structural characterization and Raman spectroscopy

To analyze the structural characteristics and strain distribution of our nanopillar devices, we used a field-emission SEM (JSM-7600F, JEOL), AFM (Park XE 15, Park systems), and Raman spectroscopy (Alpha300 M+ , WITec). The electron acceleration voltage in SEM was set to 10 kV. AFM measurement was performed on nanopillar sample in tapping mode. The scan size, resolution, and speed were set to 10 × 10 μm^2^, 512 × 512 pixel^2^ and 0.5 Hz, respectively. For Raman spectroscopy characterization, a 532-nm excitation laser was used along with a 100× objective lens. During the measurement, additional care was taken to keep the laser power low enough to avoid any heating effect. The strain-shift coefficient of 65.4 cm^–1^/%^26^ was used to estimate the maximum strain of 1.3% in our nanopillar device. The theoretical limit of spatial resolution (i.e., diffraction-limited spatial resolution) of our Raman system can be calculated using Abbe’s diffraction limit:

2 $${{{{{\rm{Spatial}}}}}}\,{{{{{\rm{resolution}}}}}}=\,\frac{0.61\lambda }{{{{{{\rm{NA}}}}}}},$$

where *λ* is the wavelength of the excitation laser, and NA is the numerical aperture of the microscope objective. In our experiment, we used a 532-nm laser with a 0.90/100x objective lens, resulting in a spatial resolution limit of 361 nm.

### Femtosecond pump–probe measurement

Femtosecond pump–probe measurement was performed in reflection geometry. The sample was held in a closed-cycle helium cryostat (Cryostation s50, Montana Instrument). The pump pulses were generated from a femtosecond laser with a 230-fs pulse duration (CARBIDE, Light Conversion). The differential reflectance change was measured with a probe pulse (*λ*_probe_ = 1450 nm) centered at the same spatial position. The spot size of pump and probe pulses were approximately 100 × 100 μm^2^ and 10 × 10 μm^2^, respectively. A pump fluence was approximately 0.4–1 mJ cm^–2^, which was adjusted by a continuously variable neutral density filter.

### Theoretical modeling of carrier dynamics under the influence of pseudo-magnetic fields

The main carrier dynamics mechanism we present in this study can be described by the Boltzmann-like scattering equation^57^:

3 $$\frac{d{\rho }_{i}(t)}{{dt}}={{\Gamma }}_{i}^{{{{{{\rm{in}}}}}}}(t)(1-{\rho }_{i}(t))-{{\Gamma }}_{i}^{{{{{{\rm{out}}}}}}}(t){\rho }_{i}(t),$$

where the scattering rate ${{\Gamma }}_{i}^{{{{{{\rm{in}}}}}}/{{{{{\rm{out}}}}}}}\,=\,{{\Gamma }}_{i}^{{{{{{\rm{cc}}}}}}-{{{{{\rm{in}}}}}}/{{{{{\rm{out}}}}}}}\,+\,{{\Gamma }}_{i}^{{{{{{\rm{ph}}}}}}-{{{{{\rm{in}}}}}}/{{{{{\rm{out}}}}}}}$ dominates the whole thermalization process, which contains two parts: pure carrier–carrier scattering ${{\Gamma }}_{i}^{{{{{{\rm{cc}}}}}}-{{{{{\rm{in}}}}}}/{{{{{\rm{out}}}}}}}$ and carrier–optical phonon scattering ${{\Gamma }}_{i}^{{{{{{\rm{ph}}}}}}-{{{{{\rm{in}}}}}}/{{{{{\rm{out}}}}}}}$. The in-scattering rate ${{\Gamma }}_{i}^{{{{{{\rm{in}}}}}}}$ and out-scattering rate ${{\Gamma }}_{i}^{{{{{{\rm{out}}}}}}}$ contain all the contributions from and into other allowed Landau levels, respectively. The explicit forms of Coulomb interaction-induced scattering rates can be written as^32,57^:

4 $${{\Gamma }}_{f}^{{{{{{\rm{cc}}}}}}-{{{{{\rm{in}}}}}}}(t)=\frac{2{{{{{\rm{\pi }}}}}}}{\hslash }\mathop{\sum}\limits_{{abc}}{V}_{{bc}}^{{fa}}({V}_{{fa}}^{{bc}}-{V}_{{fa}}^{{cb}})(1-{\rho }_{a}){\rho }_{b}{\rho }_{c}{L}_{\gamma }(\Delta {E}_{{bc}}^{{fa}}),$$

5 $${\Gamma}_{i}^{{{{\rm{cc}}}}-{{{\rm{out}}}}}(t)=\frac{2{{{\rm{\pi}}}}}{\hslash }\mathop{\sum}\limits_{{abc}}{V}_{{bc}}^{{ia}}({V}_{{ia}}^{{bc}}-{V}_{{ia}}^{{cb}}){\rho }_{a}(1-{\rho }_{b})(1-{\rho }_{c}){L}_{\gamma }(\Delta {E}_{{bc}}^{{ia}}),$$

where ${V}_{{bc}}^{{ia}}$ is the Coulomb matrix element calculated by the graphene Landau level basis, ${\rho }_{a}$, ${\rho }_{b}$ and ${\rho }_{c}$are the populations of all the possible Landau level states, and ${L}_{\gamma }(\varDelta {E}_{{bc}}^{{ia}})$ is the Lorentzian for the energy conservation in the scattering. $\varDelta {E}_{{bc}}^{{ia}}\,=\,{E}_{b}\,-\,{E}_{i}\,+\,{E}_{c}\,-\,{E}_{a}$ is the energy difference between initial states (*b, c*) and final states (*a, i*). For the carrier–optical phonon scattering part, the scattering rate can be written as^32^:

6 $${{\Gamma }}_{i}^{{{{{{\rm{ph}}}}}}-{{{{{\rm{in}}}}}}}(t)=\frac{2{{{{{\rm{\pi }}}}}}}{\hslash }\mathop{\sum}\limits_{{j},{{{{{\bf{p}}}}}},\mu }{|{G}_{{ij}}^{{{{{{\bf{p}}}}}}\mu }|}^{2}{\rho }_{j}(({n}_{{{{{{\bf{p}}}}}}\mu }+1){L}_{\gamma }(\Delta {E}_{{ij}\mu }^{{em}})+{n}_{{{{{{\bf{p}}}}}}\mu }{L}_{\gamma }(\Delta {E}_{{ij}\mu }^{{ab}})),$$

7 $${{\Gamma }}_{i}^{{{{{{\rm{ph}}}}}}-{{{{{\rm{out}}}}}}}(t)=\frac{2\pi }{\hslash }\mathop{\sum}\limits_{{j},{{{{{\bf{p}}}}}},\mu }{|{G}_{{ij}}^{{{{{{\bf{p}}}}}}\mu }|}^{2}(1\,-\,{\rho }_{{j}})(({n}_{{{{{{\bf{p}}}}}}\mu }+1){L}_{\gamma }(\Delta {E}_{{ij}\mu }^{{em}})+{n}_{{{{{{\bf{p}}}}}}\mu }{L}_{\gamma }(\Delta {E}_{{ij}\mu }^{{ab}})),$$

where ${\rho }_{j}$ is all the possible allowed states for the carrier–optical phonon scattering process, ${n}_{{{{{{\bf{p}}}}}}\mu }$ is the population of the ${\mu }$-mode phonon with wave vector *p*, which is determined by Bose–Einstein distribution, and ${L}_{\gamma }(\varDelta {E}_{{ij}{{{{{\rm{\mu }}}}}}}^{{em}})$ is the Lorentzian for the energy conservation. In the carrier–optical phonon scattering process, electrons can raise their energy levels by absorbing phonons (dominated by the term ${\rho }_{j}({n}_{{{{{{\bf{p}}}}}}\mu }\,+\,1){L}_{\gamma }(\varDelta {E}_{{ij}{{{{{\rm{\mu }}}}}}}^{{em}})$) or lower their energy levels by emitting phonons (dominated by the term ${\rho }_{j}{n}_{{{{{{\bf{p}}}}}}\mu }{L}_{\gamma }(\varDelta {E}_{{{{{{\rm{i}}}}}}{j}{{{{{\rm{\mu }}}}}}}^{{ab}})$). Considering the ambient temperatures of our study (4 K and 300 K), the population ${n}_{p\mu }$of the optical phonon is very small, which makes the phonon emission as the main process.

## Supplementary information

Supplementary Information

## Data Availability

All relevant data are available from the corresponding authors upon reasonable request.
