# Supplementary material for: Pseudo-magnetic field-induced slow carrier dynamics in periodically strained graphene
Source: Nat Commun. 2021 Aug 24;12:5087. doi: 10.1038/s41467-021-25304-0 (PMC8384878; doi:10.1038/s41467-021-25304-0)
Supplement: Supplementary file 1 — Supplementary Information [file 41467_2021_25304_MOESM1_ESM.pdf]

Supplementary Information

Pseudo-magnetic field-induced slow carrier dynamics in periodically strained graphene

Dong-Ho Kang[1†], Hao Sun[1†], Manlin Luo[1†], Kunze Lu[1], Melvina Chen[1], Youngmin Kim[1], Yongduck Jung[1], Xuejiao Gao[1], Samuel Jior Parluhutan[1], Junyu Ge[2], See Wee Koh[2], David Giovanni[3], Tze Chien Sum[3], Qi Jie Wang[1,3], Hong Li[2] and Donguk Nam[1*]

[1]School of Electrical and Electronic Engineering, Nanyang Technological University, 50 Nanyang Avenue, Singapore 639798, Singapore
[2]School of Mechanical and Aerospace Engineering, Nanyang Technological University, 50 Nanyang Avenue, Singapore 639798, Singapore
[3]Division of Physics and Applied Physics, School of Physical and Mathematical Sciences, Nanyang Technological University, 21 Nanyang Link, Singapore 637371, Singapore

[†]These authors contributed equally to this work.
[*]E-mail: dnam@ntu.edu.sg

Table of Content

Supplementary Note 1. Fabrication procedure

Supplementary Note 2. Raman analysis on unstrained and strained graphene

Supplementary Note 3. Calculation of local strain distribution for strained graphene nanopillars

Supplementary Note 4. Calculation of pseudo-magnetic fields for strained graphene nanopillars

Supplementary Note 5. Calculation of local density of states (LDOS) of strained graphene nanopillars

Supplementary Note 6. Theoretical modeling for the pseudo-magnetic field effect on carrier dynamics

Supplementary Note 7. Theoretical modelling for the carrier dynamics under pseudo-magnetic fields with carrier–carrier and carrier–optical phonon scatterings

Supplementary Figures 1–12

References

Supplementary Note 1. Fabrication procedure

Fabrication of a nanostructured substrate: Supplementary Figure 1 illustrates the fabrication procedure for a nanostructured substrate. We first spin-coated a polymethyl methacrylate (PMMA) resist (950 PMMA A6, MICROCHEM) on a 300-nm-thick SiO_2/Si substrate at 4500 rpm for 30 sec, followed by baking at 180 ºC for 2 min (Supplementary Figs. 1a–b). Then, an etching mask (an array with 1-µm diameter holes formed at 1.6-µm intervals) was patterned (Supplementary Fig. 1c) using Raith e-line e-beam lithography system. The substrate was then immersed in buffered oxide etch (BOE) (12.5% HF, 87.5% NH_4F) with 3 min 30 sec at room temperature (Supplementary Fig. 1d). PMMA resist was removed by acetone (80 ºC, 10 min), isopropyl alcohol (IPA) (room temperature, 2 min), deionized (DI) water (room temperature, 2 min), and O_2 plasma treatment (150 W, 3 min), followed by atomic layer deposition (ALD) to deposit a 20-nm Al_2O_3 layer on the entire substrate (Supplementary Figs. 1e–f).

Graphene wet transfer process: Supplementary Figure 2 shows the detailed schematic illustration for graphene wet transfer process. A PMMA supporting layer (950 PMMA A6, MICROCHEM) was spin-coated onto a graphene/SiO_2/Si substrate at 4500 rpm for 90 sec, followed by baking at 180 ºC for 2 min (Supplementary Figs. 2a–b). A high-quality large-area chemical vapor deposition (CVD)-grown monolayer graphene was used in our study. The PMMA-covered graphene layer was floated by 5% diluted hydrofluoric acid (HF) solution by etching away the underlying SiO_2 layer. After the PMMA/graphene layer was lifted off in the HF solution, it was transferred to DI water to remove HF residue. The nanostructured substrate was then used to fish the PMMA/graphene layer (Supplementary Fig. 2c). The sample was then dried at room temperature while standing at an angle to make a tight adhesion between graphene and the nanostructured substrate by capillary force[1] (Supplementary Fig. 2d). Afterwards, the PMMA resist was removed by acetone, IPA, and DI water (Supplementary Fig. 2e).

Supplementary Note 2. Raman analysis on unstrained and strained graphene

The Raman spectra of unstrained and strained graphene fabricated using the previously described procedure are both plotted in Supplementary Fig. 3. Supplementary Figure 3a compares the Raman spectra of the unstrained and strained graphene near the G mode peak, which arises from the doubly degenerate zone center phonons at Γ[2]. Similar to the 2D peak, tensile strain in the nanopillar causes the G peak to red-shift away from the unstrained graphene peak at 1581.9 cm^{-1}. Furthermore, strain breaks the rotation symmetry of the zone center phonons[3], creating G-peak splitting with two peaks at 1544.87 cm^{-1} and 1574.7 cm^{-1}. The 2D peak shown in Supplementary Fig. 3b, arising from the second order scattering of the zone-boundary phonons, has a peak value of 2691.7 cm^{-1} in unstrained graphene. By applying tensile strain, the graphene C-C bonds are elongated and become weaker, causing the 2D peak to red-shift. The splitting of the two peaks into 2606.5 cm^{-1} and 2674.3 cm^{-1} shows that the C-C bonds are being stretched by varying degrees[4].

Supplementary Note 3. Calculation of local strain distribution for strained graphene nanopillars

Structural distortion-induced strain in graphene nanopillars can be evaluated using the strain tensors, which are characterized as[5]:

$$\epsilon_{ij}(\boldsymbol{r}) = \frac{1}{2}\left(\partial_i u_j(\mathbf{r}) + \partial_j u_i(\mathbf{r}) + \partial_i h(\mathbf{r})\partial_j h(\mathbf{r})\right), (1)$$

where $\mathbf{u(r)}$ and $h(\mathbf{r})$ represent the in-plane and out-of-plane deformation fields, respectively. We mainly focus on the out-of-plane deformation induced by nanopillars, since the resulting in-plane lattice distortion is much smaller than the vertical lattice distortion[6]. Consequently, from given atomic structural distortions of graphene, which can be determined by high-resolution atomic force microscopy (AFM), the strain components can be calculated as:

$$\epsilon_{xx} = \frac{1}{2}\left(\frac{\partial h(\mathbf{r})}{\partial x}\right)^2, (2)$$

$$\epsilon_{yy} = \frac{1}{2}\left(\frac{\partial h(\mathbf{r})}{\partial y}\right)^2, (3)$$

$$\epsilon_{xy} = \frac{1}{2}\frac{\partial h(\mathbf{r})}{\partial x}\frac{\partial h(\mathbf{r})}{\partial y}, (4)$$

where $h(\mathbf{r})$ is the AFM topographic data, and $\mathbf{r}$ denotes the position in the x-y plane. The resulting strain distributions are presented in Fig. 2c, which are also used to calculate the spatial distribution of pseudo-magnetic fields as explained in Supplementary Note 4.

Supplementary Note 4. Calculation of pseudo-magnetic fields for strained graphene nanopillars

The Hamiltonian modified by pseudo-gauge fields induced by strain in wave-vector space can be written as[7]:

$$H(\mathbf{q}) = v_0\boldsymbol{\sigma}\cdot(\mathbf{q} + \mathbf{A}), \quad (5)$$

where $v_0 = \frac{3a_0t_0}{2}$ is the Fermi velocity, and the typical value is about 10^6 ms^{-1}, $a_0 = 0.14$ nm is the length of the carbon-carbon bond, $t_0 = 2.7$ is the hopping amplitude in the tight-binding model. $\boldsymbol{\sigma}$ is the 2 × 2 Pauli matrix for the sublattice freedom. The pseudo-gauge field or vector potential $\mathbf{A}$ arising from the strain effect is expressed as[5,7]:

$$A_1 = \frac{\beta}{2a_0}(\epsilon_{11} - \epsilon_{22}), \quad A_2 = \frac{\beta}{2a_0}(-2\epsilon_{12}), \quad (6)$$

Where $\beta \approx 3$ is a constant. Since strain does not break the time reversal symmetry, the vector potential $\mathbf{A}$ will possess opposite signs for the two inequivalent $\mathbf{K}$ and $\mathbf{K}'$ points, making the net magnetic field to be zero. The pseudo-magnetic field perpendicular to the surface is given by $\mathbf{B} = \nabla \times \mathbf{A}$ and can be written as a function of the strain tensors[7]:

$$B_S = \frac{-\beta}{2a_0}\left(\partial_y\epsilon_{xx}(x,y) - \partial_y\epsilon_{yy}(x,y) + 2\partial_x\epsilon_{xy}(x,y)\right). \quad (7)$$

One can see that the pseudo-magnetic field, B_S, vanishes for a uniform strain and that only non-uniform strain contributes to the finite value of the induced field strength. When the pseudo-magnetic field is relatively uniform under a specific strain field (e.g., a well-designed triaxial strain), the Dirac bands can be rearranged into non-equidistant pseudo-Landau levels having energies of E_n, which can be approximated as:

$$E_n = \mathrm{sgn}(n)\hbar v_0\sqrt{\frac{2e_0}{\hbar}|nB_S|}, \quad (8)$$

where n is the Landau level index and e_0 is the electron charge. One can insert the values of all constants, and obtain a simplified one:

$$E_n = 36\mathrm{sgn}(n)\sqrt{|nB_S|}\ (\mathrm{meV}). \quad (9)$$

Supplementary Note 5. Calculation of local density of states (LDOS) of strained graphene nanopillars

LDOS with different pseudo-magnetic fields can be calculated by the following expression:

$$\mathrm{DOS}(E) = \frac{1}{\pi}\sum_n \frac{\gamma}{(E-E_n)^2+\gamma^2}, \quad (10)$$

where γ is the broadening factor of Landau level. Supplementary Figure 4 shows the calculated LDOS for the strained graphene with different pseudo-magnetic fields (10, 40, and 80 T).

Supplementary Note 6. Theoretical modeling for the pseudo-magnetic field effect on carrier dynamics

6.1 Classic light-matter interaction and pumping pulse

We consider a classic light-carrier coupling for pumping process. The excitation pulse can be written as[8]:

$$\mathbf{A}(t) = A_{\mathrm{env}}\left(A_0^+\begin{pmatrix}\cos(\omega t)\\ \sin(\omega t)\end{pmatrix} + A_0^-\begin{pmatrix}\cos(\omega t)\\ \text{-}\sin(\omega t)\end{pmatrix}\right), \quad (11)$$

where A_{env} is the envelope function that has the following form:

$$A_{\mathrm{env}} = \frac{1}{\omega}\sqrt{\frac{2\sqrt{\ln 2}e_{\mathrm{pf}}}{\sqrt{\pi}\epsilon_0 c\sigma_{\mathrm{FWHM}}}}e^{-\frac{2\ln 2t^2}{\sigma_{\mathrm{FWHM}}^2}}, \quad (12)$$

where e_{pf} is the pump fluence, ω is the frequency of pump light, ϵ_0 is the dielectric constant, and σ_{FWHM} is the full width at half maximum (FWHM) of the pump light.

6.2 Quantum mechanical description of many-particle system

The full many-particle Hamiltonian with electron, phonon and photon parts in our system can be expressed as:

$$H = H_0 + H_{\text{e-e}} + H_{\text{e-ph}} + H_{\text{e-pt}}, \quad (13)$$

Each term has the following expressions:

$$H_0 = \sum_i \epsilon_i a_i^\dagger a_i + \sum_{v\mathbf{q}} \hbar\Omega_{v\mathbf{q}} b_{v\mathbf{q}}^\dagger b_{v\mathbf{q}} + \sum_\mu \hbar\omega_\mu c_\mu^\dagger c_\mu, \quad (14)$$

$$H_{\text{e-e}} = \frac{1}{2}\sum_{ijkl} v_{kl}^{ij} a_i^\dagger a_j^\dagger a_k a_l, \quad (15)$$

$$H_{\text{e-ph}} = \sum_{ijv\mathbf{q}} G_{ij}^{v\mathbf{q}} a_i^\dagger a_j \left(b_{v\mathbf{q}} + b_{\text{-}\mathbf{q}v}^\dagger\right), \quad (16)$$

$$H_{\text{e-pt}} = i\hbar\sum_{ij\mu}\left(g_{ij}^\mu a_i^\dagger a_j c_\mu \text{-} g_{ij}^{\mu *} a_j^\dagger a_i c_\mu^\dagger\right) - \frac{i\hbar e_0}{m_0}\sum_{ij}\mathbf{M}_{ij}\cdot\mathbf{A}(t) a_i^\dagger a_j, \quad (17)$$

where $a_i^\dagger$, $b_{v\mathbf{q}}^\dagger$, $c_\mu^\dagger$ are the electron, phonon, and photon creation operators, ϵ_i is the single-particle energy for electron, v_{kl}^{ij} is the Coulomb matrix element, $\mathbf{M}_{ij}$ is the optical matrix element, $\mathbf{A}$ is the vector potential of the external pump light, $\Omega_{v\mathbf{q}}$ is the frequency of phonon, $G_{ij}^{v\mathbf{q}}$ (g_{ij}^μ) is the electron–phonon (electron–photon) coupling matrix element, and ω_μ is the frequency of photon[9].

6.3 Optical Bloch equation

We study the carrier dynamics using the optical Bloch equations (OBE). With the Heisenberg equation of motion, the time evolution of the electron population can be described by[8]:

$$\frac{d\rho_l}{dt} = 2\mathrm{Re}\left(\Sigma_i \Omega_{fi} p_{il}\right)$$
$$\frac{dp_{if}}{dt} = \left(i\omega_{if} - \gamma\right)p_{if} + \Omega_{fi}\left(\rho_f - \rho_i\right) \quad (18)$$

where $\rho_l(t) = \langle a_l^\dagger a_l \rangle(t)$ is the electron population at a pseudo-Landau level with quantum index l, p_{if} is the microscopic polarization, Ω_{fi} is the Rabi frequency, and $\omega_{if} = \frac{E_f - E_i}{\hbar}$ is the frequency difference between initial and final states. The change of the electron populations for the probe energy (i.e., $\Delta\rho_f - \Delta\rho_i$) as a function of time is directly proportional to the measured reflection change ($\Delta R/R$) presented in our study[10]. The electron–electron Coulomb scattering is the main factor that dominates the non-equilibrium carrier dynamics and is considered for the many-particle interactions in the OBE. The electron–electron Coulomb interaction is written as[8]:

$$H_{\text{e-e}} = \frac{1}{2}\sum_{ijkl} v_{ij}^{kl} a_i^\dagger a_j^\dagger a_k a_l. \quad (19)$$

The many-particle scattering rate by the electron–electron interactions can be written by:

$$\Gamma_f^{\text{cc,in}}(t) = \frac{2\pi}{\hbar}\sum_{abc} V_{bc}^{fa}\left(V_{fa}^{bc} - V_{fa}^{cb}\right)\left(1 - \rho_a\right)\rho_b\rho_c L_\gamma\left(\Delta E_{bc}^{fa}\right), \quad (20)$$

$$\Gamma_i^{\text{cc,out}}(t) = \frac{2\pi}{\hbar}\sum_{abc} V_{bc}^{ia}\left(V_{ia}^{bc} - V_{ia}^{cb}\right)\rho_a\left(1 - \rho_b\right)\left(1 - \rho_c\right)L_\gamma\left(\Delta E_{bc}^{ia}\right), \quad (21)$$

where V_{bc}^{ia} is the Coulomb matrix element. It can be written as:

$$V_{bc}^{ia} = \sum_{\mathbf{q}} V(\mathbf{q})\rho_{ib}(\mathbf{q})\rho_{ac}(-\mathbf{q}) = \alpha_{n_b n_c}^{n_i n_a}\delta_{\xi_i,\xi_b}\delta_{\xi_a,\xi_c} c_{bc}^{ia}\frac{e_0}{\epsilon_0}\int d\mathbf{q}\frac{1}{\mathbf{q}}\widetilde{F}_b^i(\mathbf{q})\widetilde{F}_c^a(\mathbf{q}), \quad (22)$$

where $\widetilde{F}_b^i(\mathbf{q})$ is the combined form factor, which considers the shape of different Landau levels:

$$\widetilde{F}_b^i(\mathbf{q}) = \mathrm{sgn}(n_i n_b)F_{n_b\text{-}1,m_b}^{n_i\text{-}1,m_i}(\mathbf{q}) + F_{n_b,m_b}^{n_i,m_i}(\mathbf{q}). \quad (23)$$

$F_{n_b\text{-}1,m_b}^{n_i\text{-}1,m_i}(\mathbf{q})$ is the general form factor[11], and the coefficient $\alpha_{n_b n_c}^{n_i n_a}$ is written as:

$$\alpha_{n_b n_c}^{n_i n_a} = \left(\sqrt{2}\right)^{\delta_{n_i,0}+\delta_{n_a,0}+\delta_{n_b,0}+\delta_{n_c,0}}, \quad (24)$$

and c_{bc}^{ia} is a constant. Lorentzian $L_\gamma\left(\Delta E_{bc}^{ia}\right)$ for the conservation of energy is defined as:

$$L_\gamma(\Delta E) = \frac{1}{\pi}\frac{\gamma}{\Delta E^2 + \gamma^2}, \quad (25)$$

where $\Delta E_{bc}^{ia} = E_b - E_i + E_c - E_a$. Due to the presence of many electrons and the surrounding material, the Coulomb potential will be screened and renormalized as[8]:

$$v_q \rightarrow \frac{v_q}{\epsilon_r(\mathbf{q},\omega)\epsilon_b}. \quad (26)$$

The dielectric function $\epsilon_r(\mathbf{q},\omega)$ can be calculated as:

$$\epsilon_r(\mathbf{q},\omega) = 1 - \frac{V_q}{\epsilon_b}\Pi(\mathbf{q},\omega), \quad (27)$$

where ϵ_b is the background dielectric constant, $\Pi(\mathbf{q},\omega)$ is the polarizability calculated in the random phase approximation (RPA) according to Goerbig *et al.*[12] Thus, the Coulomb interaction-modified OBEs now read as[8]:

$$\frac{d\rho_l}{dt} = \Gamma_l^{\text{cc,in}}\left(1-\rho_l\right) - \Gamma_l^{\text{cc,out}}\rho_l$$
$$\frac{dp_{if}}{dt} = \frac{1}{2}\left(\Gamma_i^{\text{cc,in}} + \Gamma_i^{\text{cc,out}} + \Gamma_f^{\text{cc,in}} + \Gamma_f^{\text{cc,out}}\right)p_{if} \quad (28)$$

We point out that the main many-body interactions considered in the rise process are electron–electron scattering, in which the photoexcited carrier distribution rapidly broadens as pairs of electrons scatter to lower and higher energy states. Since the pump energy is larger than the probe energy, we assume that the dynamical picture of the rise process is that the excited carriers are first pumped to the initial Landau level, LL_i, and then inject to a lower energy level that is the final Landau level, LL_f, through electron–electron scattering channels. The experimentally measured probe signals indicate the time of carrier filling in the target LL_f level. In Fig. 4, the calculated probe rise times for the target LL_f level show a clear slow-down of carrier relaxation when the pseudo-magnetic field intensity is increased from 20 to 80 T (4 cases: 20 T, 40 T, 60 T, 80 T). Based on the pump (1030 nm) and probe energies (1450 nm), the target levels are chosen differently for different pseudo-magnetic fields. In principle, the dominated electron–electron scattering requires the energy and momentum conservation, but the non-equidistant energetic separation feature of the pseudo-Landau levels in strained graphene can effectively suppress the carrier scattering because energy conservation is not fulfilled[13]. This is in fact in contrast to the data for unstrained graphene having a short rise time of a few tens of femtoseconds owing to the existence of ample electron–electron scattering channels within the continuous Dirac

bands. A larger pseudo-magnetic field and a larger separation of pseudo-Landau levels lead to a longer time for carriers to relax from the pump energy level to the probe energy level, which is highly consistent with our experimental observation (Fig. 3).

Supplementary Note 7. Theoretical modelling for the carrier dynamics under pseudo-magnetic fields with carrier–carrier and carrier–optical phonon scatterings

The main carrier dynamics mechanism we present in this study can be described by the Boltzmann-like scattering equation[14]:

$$\frac{d\rho_i(t)}{dt} = \Gamma_i^{\text{in}}(t)\left(1-\rho_i(t)\right) - \Gamma_i^{\text{out}}(t)\rho_i(t) \quad (29)$$

where the scattering rate $\Gamma_i^{\text{in/out}} = \Gamma_i^{\text{cc-in/out}} + \Gamma_i^{\text{ph-in/out}}$ dominates the whole thermalization process, which contains two parts: pure carrier–carrier scattering $\Gamma_i^{\text{cc-in/out}}$ and carrier–optical phonon scattering $\Gamma_i^{\text{ph-in/out}}$. The in-scattering rate Γ_i^{in} and out-scattering rate Γ_i^{out} contain all the contributions from and into other allowed Landau levels, respectively. The explicit forms of Coulomb interaction-induced scattering rates can be written as[8,14]:

$$\Gamma_f^{\text{cc-in}}(t) = \frac{2\pi}{\hbar}\sum_{abc} V_{bc}^{fa}\left(V_{fa}^{bc} - V_{fa}^{cb}\right)\left(1-\rho_a\right)\rho_b\rho_c L_\gamma\left(\Delta E_{bc}^{fa}\right)$$

$$\Gamma_i^{\text{cc-out}}(t) = \frac{2\pi}{\hbar}\sum_{abc} V_{bc}^{ia}\left(V_{ia}^{bc} - V_{ia}^{cb}\right)\rho_a\left(1-\rho_b\right)\left(1-\rho_c\right)L_\gamma\left(\Delta E_{bc}^{ia}\right), \quad (30)$$

where V_{bc}^{ia} is the Coulomb matrix element calculated by the graphene Landau level basis, ρ_a, ρ_b and ρ_c are the populations of all the possible Landau level states, and $L_\gamma(\Delta E_{bc}^{ia})$ is the Lorentzian for the energy conservation in the scattering. $\Delta E_{bc}^{ia} = E_b - E_i + E_c - E_a$ is the energy difference between initial states $(b,\ c)$ and final states $(a,\ i)$.

In pristine graphene, it is possible to achieve thermalized carrier distribution within 200 fs by efficient Auger processes (i.e., Auger recombination and impact ionization) enabled by carrier–carrier scattering[14]. However, in Landau quantized graphene system with pseudo-magnetic fields, the Auger process will not be sufficient for the whole thermalization process[13]. Particularly for the higher Landau levels, the energy conservation for carrier scattering will not always be matched, leading to a very long thermalization time when only carrier–carrier interaction is considered. We therefore emphasize that optical phonon-assisted carrier relaxation is also critical to establish full thermalization.

We consider all the optical phonon modes that can influence phonon-assisted carrier depopulation, including ΓTO, ΓLO, KTO, and KLO, which have the energies of 192 meV, 198 meV, 162 meV, and 151 meV, respectively[8]. We also considered the out-of-plane optical phonon ZO mode that is relevant to the thermalization process in the higher Landau levels with small energy spacing[15]. In the calculations, the electron–optical phonon coupling matrix element for the above-mentioned phonon modes can be expressed as[8]:

$$G_{ij}^{\mathbf{p}\mu} = \int_{v} d\mathbf{r}\psi_f^* V_p^{\mu}(\mathbf{p})\psi_i = \left\langle \psi_{f,m_f}^{\xi_f} \middle| V_p^{o}(\mathbf{p}) \middle| \psi_{i,m_i}^{\xi_i} \right\rangle, \quad (31)$$

where $V_p^{o}(\mathbf{p})$ is the electron–optical phonon coupling potential with only off-diagonal elements, and $\psi_{f,m_f}^{\xi_f}$ is the wave function of the Landau-quantized state. Using coupling matrix elements, we can calculate the hot carrier thermalization assisted by optical phonon modes. The optical phonon-induced scattering rates can be written as[8]:

$$\begin{aligned} \Gamma_i^{\text{ph-in}}(t) &= \frac{2\pi}{\hbar}\sum_{j,\mathbf{p},\mu}\left|G_{ij}^{\mathbf{p}\mu}\right|^2 \rho_j \left(\left(n_{\mathbf{p}\mu}+1\right)L_\gamma\left(\Delta E_{ij\mu}^{em}\right) + n_{\mathbf{p}\mu}L_\gamma\left(\Delta E_{ij\mu}^{ab}\right)\right) \\ \Gamma_i^{\text{ph-out}}(t) &= \frac{2\pi}{\hbar}\sum_{j,\mathbf{p},\mu}\left|G_{ij}^{\mathbf{p}\mu}\right|^2 \left(1-\rho_j\right)\left(\left(n_{\mathbf{p}\mu}+1\right)L_\gamma\left(\Delta E_{ij\mu}^{em}\right) + n_{\mathbf{p}\mu}L_\gamma\left(\Delta E_{ij\mu}^{ab}\right)\right), \end{aligned} \quad (32)$$

where ρ_j is all the possible allowed states for the carrier–optical phonon scattering process, $n_{\mathbf{p}\mu}$ is the population of the μ-mode phonon with wave vector **p**, which is determined by Bose-Einstein distribution, and $L_\gamma\left(\Delta E_{ij\mu}^{em}\right)$ is the Lorentzian for the energy conservation. In the carrier–optical phonon scattering process, electrons can raise their energy levels by absorbing phonons (dominated by the term $\rho_j\left(n_{\mathbf{p}\mu}+1\right)L_\gamma\left(\Delta E_{ij\mu}^{em}\right)$), or lower their energy levels by emitting phonons (dominated by the term $\rho_j n_{\mathbf{p}\mu}L_\gamma\left(\Delta E_{ij\mu}^{ab}\right)$) Considering the ambient temperatures of our study (4 K and 300 K), the population $n_{\mathbf{p}\mu}$ of the optical phonon is very small, which makes the phonon emission as the main process.

Supplementary Figures

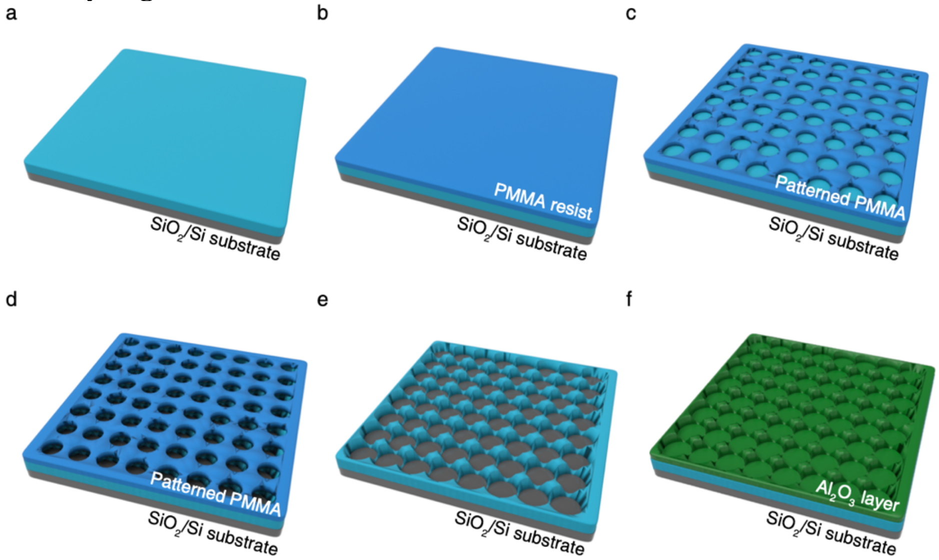

Supplementary Figure 1 | Fabrication process of a nanostructured substrate. a–b, The PMMA resist was covered on a 300-nm-thick SiO_2/Si substrate. **c,** Patterned PMMA is used as an etch mask. **d,** The sample was then soaked in BOE for 3 min 30 sec. **e–f,** The PMMA layer was removed using acetone, IPA, DI water, and O_2 plasma, followed by 20-nm-thick Al_2O_3 deposition using ALD.

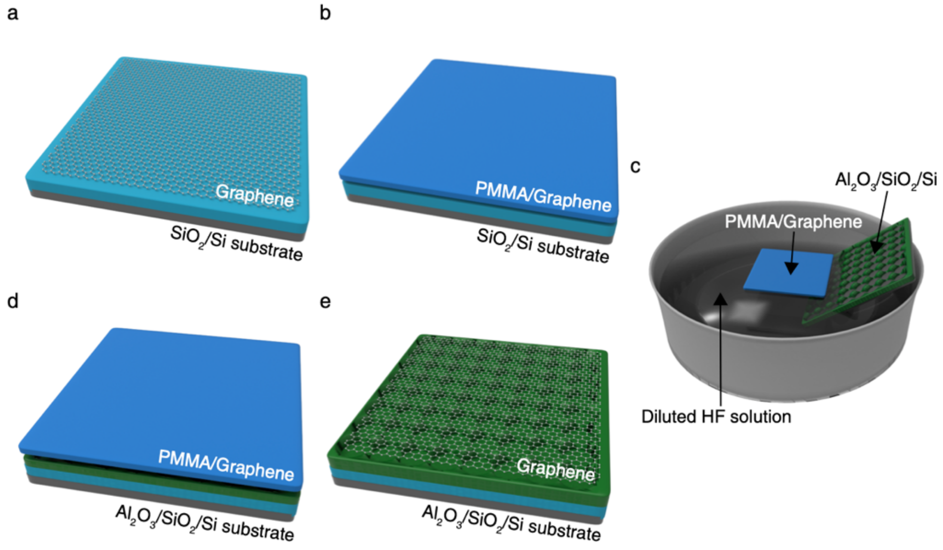

Supplementary Figure 2 | Graphene wet transfer to the nanostructured substrate. a–b, The PMMA supporting layer was covered on a graphene/SiO_2/Si sample. **c,** The PMMA/graphene layer was floated in a diluted HF solution to etch way the underlying SiO_2

layer. The PMMA/graphene layer was then transferred to DI water. The nanostructured substrate was used to fish the PMMA/graphene layer. **d,** The sample was then dried with standing at an angle to make a tight adhesion between graphene and the substrate by capillary force. **e,** PMMA was removed using acetone, IPA and DI water.

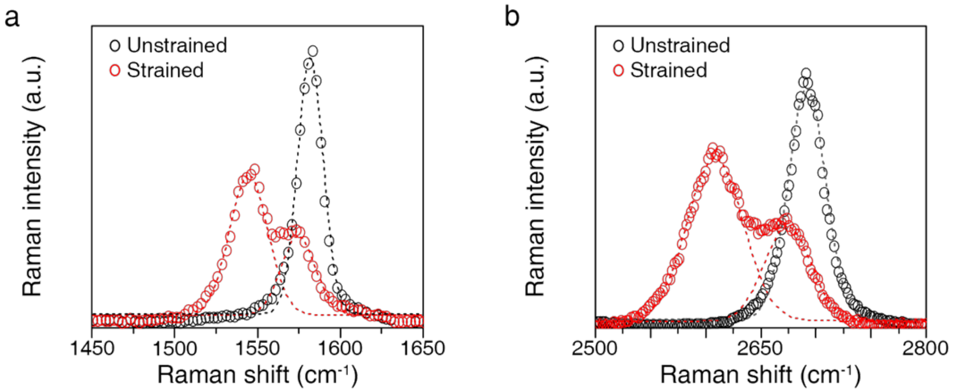

Supplementary Figure 3 | Raman analysis of unstrained and strained graphene a, Raman spectra of G mode for unstrained (black) and strained (red) graphene. **b,** Raman spectra of 2D mode for unstrained (black) and strained (red) graphene. By applying high strain on graphene using nanopillars, 2D and G peaks split into $2D^+$ / $2D^-$ and G^+ / G^-, respectively. Corresponding 2D and G peaks have been fitted with two Lorentzian peaks in each spectrum (dashed line).

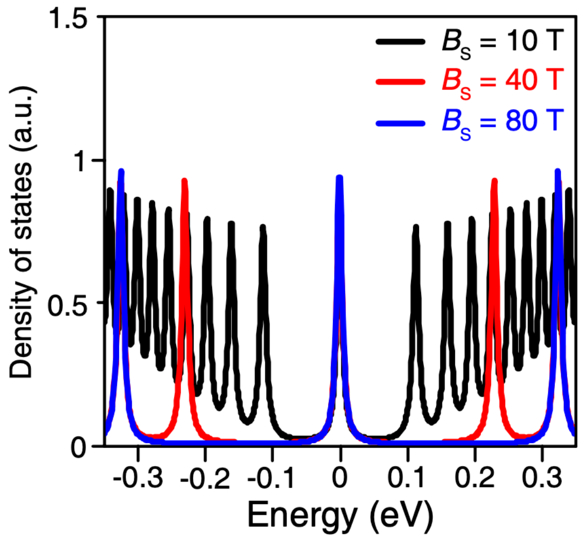

Supplementary Figure 4 | Calculated LDOS in strained graphene with 10 T (black), 40 T

(red), and 80 T (blue) of pseudo-magnetic fields. For the LDOS calculations, we employ the broadening parameter $\gamma = 7$ meV[8].

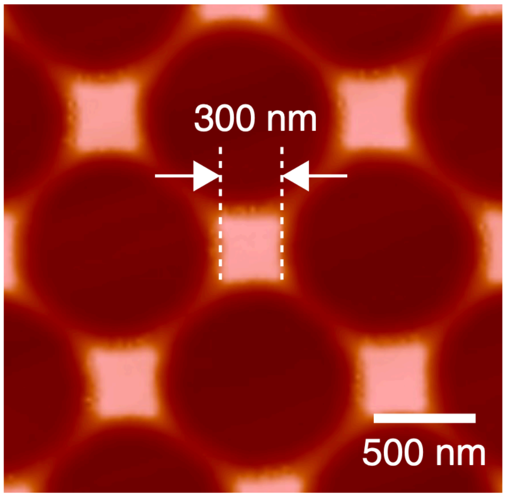

Supplementary Figure 5 | Top-view AFM image of our nanopillar array. The size of nanopillar is 300 nm.

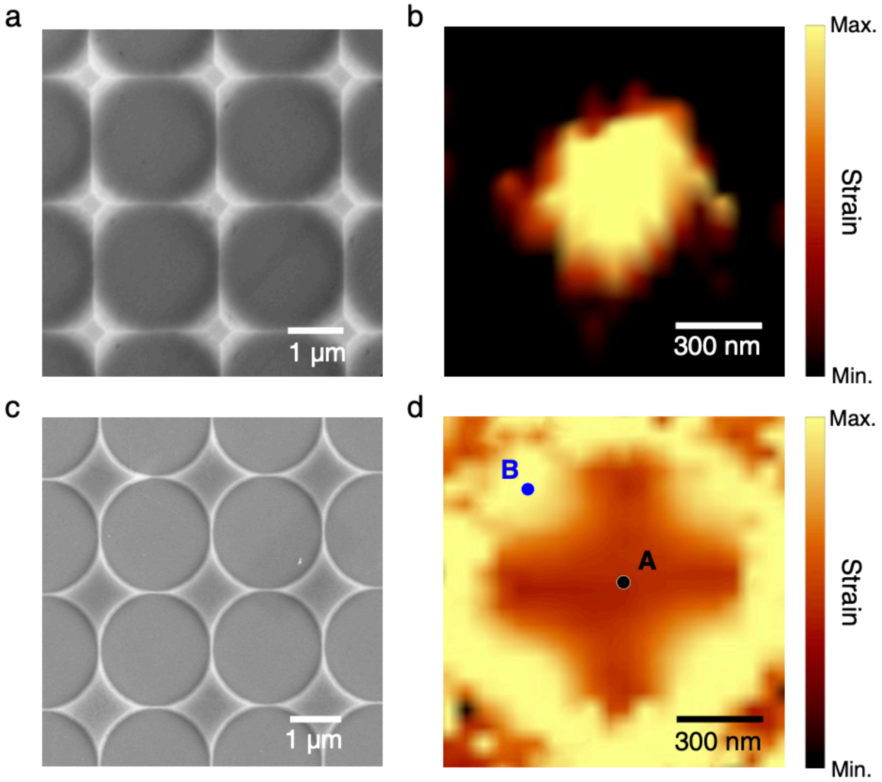

Supplementary Figure 6 | Raman measurements of strained graphene on 300-nm and

1-μm nanopillars. a) Top-view SEM image of a 300-nm nanopillar and **b)** corresponding Raman mapping image. **c)** Top-view SEM image of a 1-μm size nanopillar and **d)** corresponding Raman mapping image. The brightest regions of the Raman image (marked as B) indicate the most strained edge regions, while the darkest part (marked as A) is the less strained central part of the nanopillar.

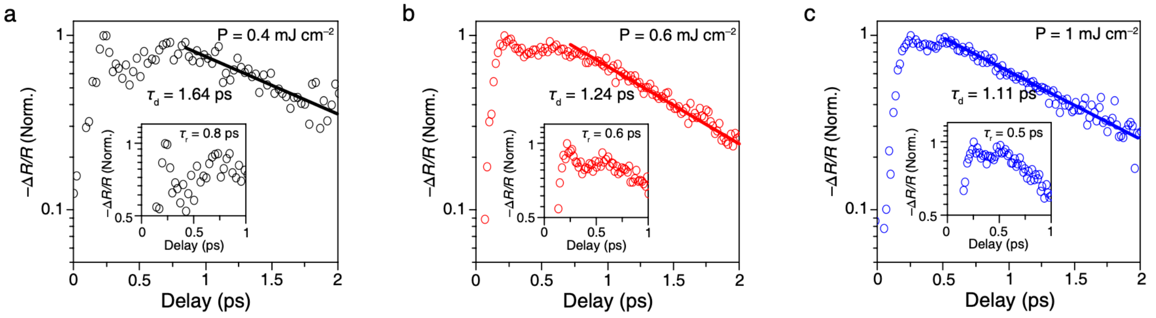

Supplementary Figure 7 | Pump-dependent carrier dynamics of the nanopillar device. Normalized reflection changes of the nanopillar sample at different pump fluence of **a,** 0.4 mJ cm^{-2}, **b,** 0.6 mJ cm^{-2}, and **c,** 1 mJ cm^{-2}. All measurements were performed at 4 K. Symbols are measurement data; lines are fitting data for decay regions. Inset: Normalized reflection changes of the nanopillar sample from 0 to 1 ps. Both rise and decay times were reduced by increasing pump fluence.

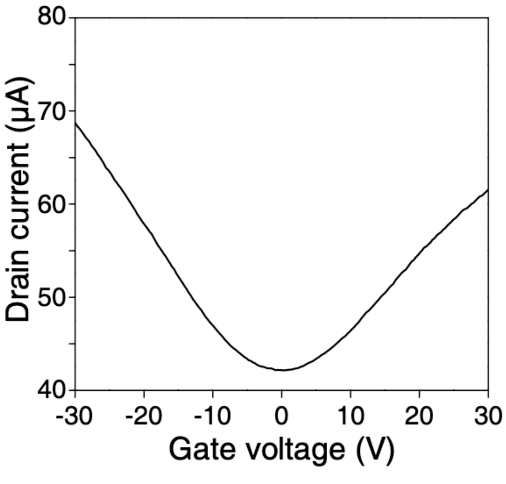

Supplementary Figure 8 | Electrical characteristic (i.e., I_{DS}–V_{GS} curve) of our monolayer graphene transistor. The Dirac point is close to 0 V, indicating that the doping

level of our monolayer graphene sheet is close to undoped.

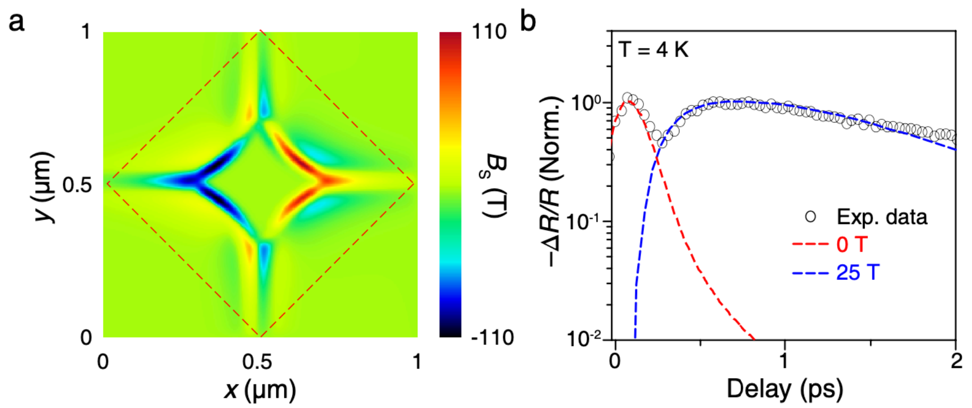

Supplementary Figure 9 | Theoretical modeling of carrier dynamics for the nanopillar device. a) Pseudo-magnetic field distribution for a unit area that contains a single graphene nanopillar. The average pseudo-magnetic field intensities for unstrained (outside of the red dashed box) and strained (inside of the red dashed box) regions are calculated to be 0 T and 25 T, respectively. **b)** Calculated carrier dynamics for unstrained (red dashed line) and strained (blue dashed line) graphene that are well matched to the experimental result shown in Fig. 3c (black empty circles).

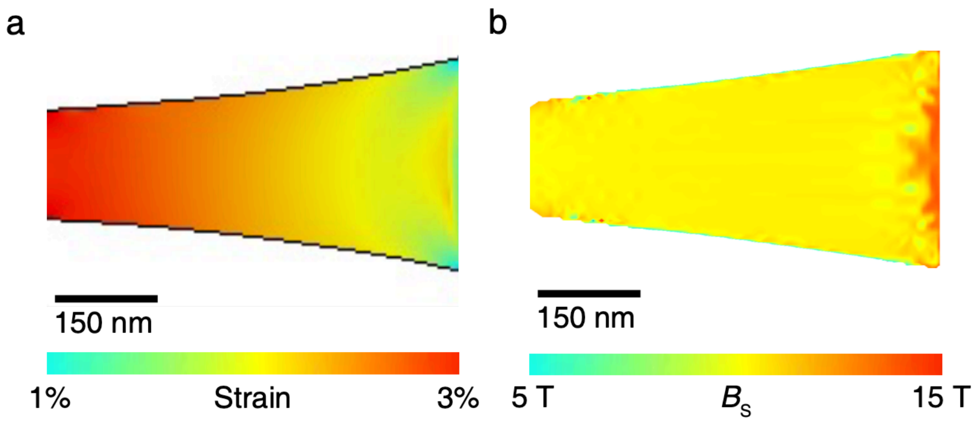

Supplementary Figure 10 | Design of uniaxial strained graphene nanowire for uniform pseudo-magnetic fields. a) Two-dimensional strain distribution and **b)** pseudo-magnetic fields distribution of a uniaxial strained graphene nanowire design.

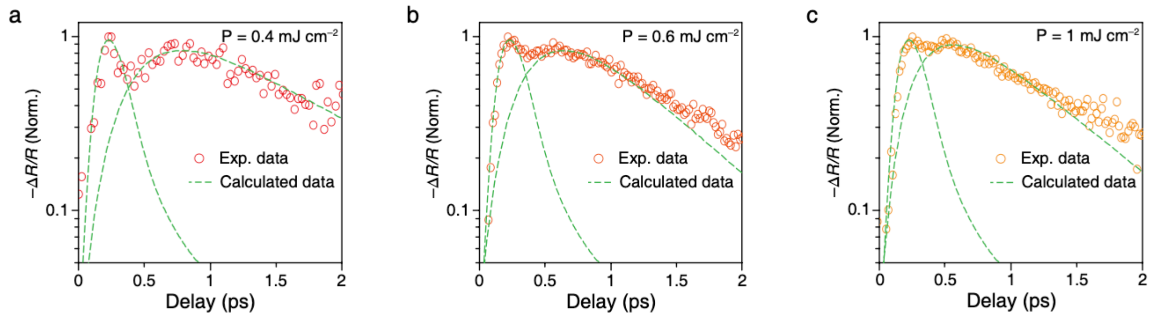

Supplementary Figure 11 | Theoretical modeling for pump fluence dependence for strained graphene nanopillar. Comparison of experimentally measured data (empty circles) with calculated electron population changes (green dashed lines) in strained graphene with the different pump fluence of **a)** 0.4 mJ cm^{-2}, **b)** 0.6 mJ cm^{-2} and **c)** 1 mJ cm^{-2}.

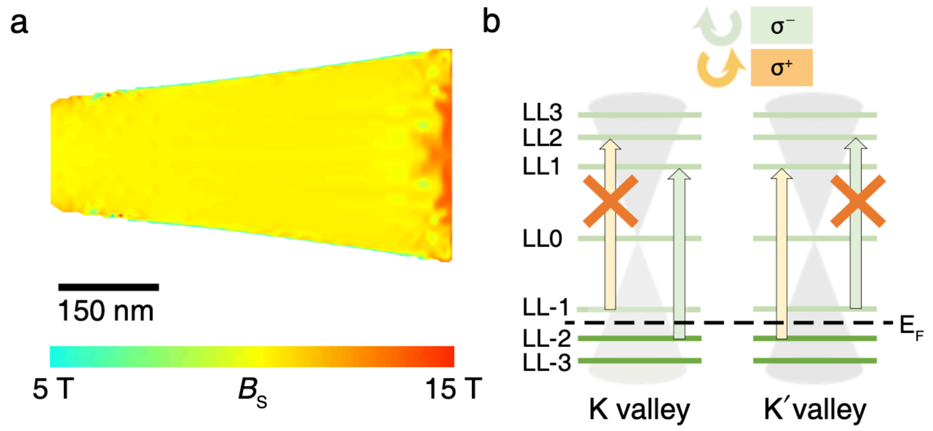

Supplementary Figure 12 | Valley-specific optical transition in strained graphene under uniform pseudo-magnetic fields. a) Pseudo-magnetic field distribution for uniaxial strained graphene and **b)** its bandstructures for both K and K′ valleys. Adjusting the Fermi level to be located between LL-1 and LL-2 can suppress the LL-1 → LL2 transition. As a result, only electrons in the K′ valley can respond to the σ^+–radiation (yellow), while electrons in the K valley can only be excited by σ^-–radiation (green).

References

1. Li, H. *et al.* Optoelectronic crystal of artificial atoms in strain-textured molybdenum disulphide. *Nat. Commun.* **6**, 7381 (2015).
2. Reichardt, S. & Wirtz, L. *Optical Properties of Graphene Ch. 3: Raman Spectroscopy of Graphene* (World Scientific Publishing, Singapore, 2017).
3. Ferrari, A. C. *et al.* Raman spectrum of graphene and graphene layers. *Phys. Rev. Lett.* **97**, 187401 (2006).
4. Yoon, D., Son, Y. W. & Cheong, H. Strain-dependent splitting of the double-resonance raman scattering band in graphene. *Phys. Rev. Lett.* **106**, 155502 (2011).
5. Guinea, F., Katsnelson, M. I. & Geim, A. K. Energy gaps and a zero-field quantum hall effect in graphene by strain engineering. *Nat. Phys.* **6**, 30–33 (2010).
6. Hsu, C. C., Teague, M. L., Wang, J. Q. & Yeh, N. C. Nanoscale strain engineering of giant pseudo-magnetic fields, valley polarization, and topological channels in graphene. *Sci. Adv.* **6**, eaat9488 (2020).
7. De Juan, F., Sturla, M. & Vozmediano, M. A. H. Space dependent Fermi velocity in strained graphene. *Phys. Rev. Lett.* **108**, 227205 (2012).
8. Wendler, F., Knorr, A. & Malic, E. Ultrafast carrier dynamics in Landau-quantized graphene. *Nanophotonics* **4**, 224–249 (2015).
9. Brem, S., Wendler, F. & Malic, E. Microscopic modeling of tunable graphene-based terahertz Landau-level lasers. *Phys. Rev. B* **96**, 045427 (2017).
10. Wendler, F. *et al.* Efficient Auger scattering in Landau-quantized graphene. *Ultrafast Phenom. Nanophotonics XIX* **9361**, 936105 (2015).
11. Haldane, F. D. M. Geometrical description of the fractional quantum hall effect. *Phys. Rev. Lett.* **107**, 116801 (2011).
12. Goerbig, M. O. Electronic properties of graphene in a strong magnetic field. *Rev. Mod. Phys.* **83**, 1193 (2011).
13. Plochocka, P. *et al.* Slowing hot-carrier relaxation in graphene using a magnetic field. *Phys. Rev. B* **80**, 245415 (2009).
14. Malic, E., Winzer, T., Bobkin, E. & Knorr, A. Microscopic theory of absorption and ultrafast many-particle kinetics in graphene. *Phys. Rev. B* **84**, 205406 (2011).

15. Politano, A., De Juan, F., Chiarello, G. & Fertig, H. A. Emergence of an out-of-plane optical phonon (ZO) Kohn anomaly in quasifreestanding epitaxial graphene. *Phys. Rev. Lett.* **115**, 075504 (2015).
